# On the origin of the Helmholtz’s square illusion: An attentional account

**DOI:** 10.3758/s13414-023-02717-1

**Published:** 2023-05-08

**Authors:** Wladimir Kirsch, Wilfried Kunde

**Affiliations:** Institut für Psychologie III der Universität Würzburg, Röntgenring 11, D-97070 Würzburg, Germany

**Keywords:** 2D shape and form, Attention, Visual perception

## Abstract

A square filled with parallel horizontal or vertical lines appears perceptually extended in the direction orthogonal to the lines. Here, we suggest that this Helmholtz illusion arises due to changes in spatial attention that entail changes at very early stages of perceptual processing. Three experiments are reported which tested this assumption. In Experiment1 and Experiment2, transient attentional cues were flashed in such a way that they either promoted (congruent condition) or hindered (incongruent condition) the attentional state presumably induced by the target objects. We predicted a decline of the illusion in the incongruent condition compared with the congruent condition. This prediction was confirmed in both experiments. However, the influence of (in)congruent attention cuing on the Helmholtz illusion depended on more sustained distributions of attention as well. An influence of sustained attention on the illusion was confirmed in Experiment 3, in which changes of attentional focus were induced by a secondary task. Overall, the results were consistent with our claim that the origin of the Helmholtz illusion is closely linked to the distribution of spatial attention.

**Supplementary Information:**

The online version contains supplementary material available at 10.3758/s13414-023-02717-1.

## Introduction

More than 150 years ago, Hermann von Helmholtz noted that a square filled with vertical lines appeared wider than taller, whereas a square filled with horizontal lines appeared taller than wider (von Helmholtz, 1867, p. 563). This apparent shape distortion has been labelled the Helmholtz illusion (or Helmholtz square illusion). The illusion has stimulated some research—interestingly, mostly regarding the question of whether correspondingly designed clothes let people look slimmer (Ashida et al., 2013; Koutsoumpis et al., 2021; Taya & Miura, 2007; Thompson & Mikellidou, 2011). Yet the factors affecting the illusion, and more importantly, its underlying perceptual processes, are surprisingly unknown. While we know that the illusion depends on the horizontal/vertical orientation of the lines, as well as their width/spacing ratio (duty cycle; Thompson & Mikellidou, 2011), it seems that typical accounts of visual illusions fall short to explain the Helmholtz illusion (e.g., Mikellidou, 2012).

The only close to mechanistic explanation we are aware of rests on Gestalt psychology (Pinna, 2015, esp. pp. 7–8). The basic idea here is that different groupings of lines cause differences in perceived shape. Grouping is supposed to be accompanied by so-called directional organization, which promotes certain shape attributes (such as a rectangle). In particular, the direction of distribution of lines gives rise to the perceived shape. That is, the perception of the square is assumed to be biased in the same direction as the lines are grouped based on their similarity. In other words, the square with horizontal/vertical lines is perceived as taller/wider because the lines are vertically/horizontally distributed and grouped in perception. The author argues that this “global” spatial orientation of line grouping is much stronger than the local direction of each line (the local line direction would accentuate an opposite shape in perception (i.e., would lead to perceiving a square with vertical lines as taller than wider and a square with horizontal lines as wider than taller).

**Fig. 1:**
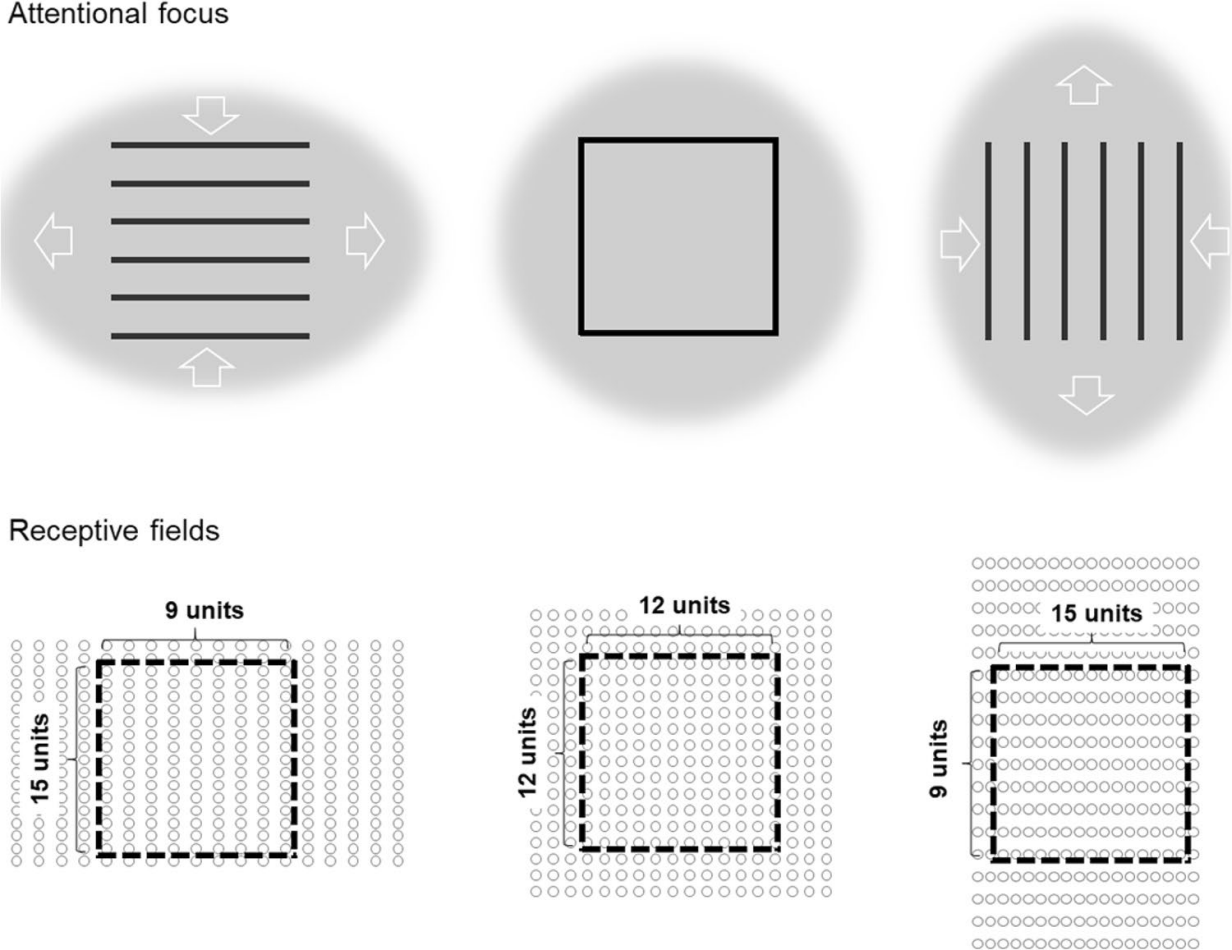
An explanation of why a square consisting of horizontal lines appears taller and narrower than a square of identical size that is composed of vertical lines (i.e., of Helmholtz illusion). The attentional field is assumed to be slightly elongated along the lines and slightly compressed in the opposite direction when compared with a “neutral” object, such as a usual square. This is indicated by white arrows, which also specify the relative shifts of receptive fields. That is, RF shift less towards the center of attention (or they shift more in the opposite direction) along the horizontal/vertical dimension for the square with horizontal/vertical lines. As a result, the RF density along the lines decreases and the square activates less neurons coding neighboring retinal location along this dimension. Provided that RF are assigned to a fixed spatial scale the Helmholtz illusion should naturally arise. For example, assume a usual attended square activates 12 neighboring columns of neurons that signal a width of the object of 12 units (see middle part of the figure). When the RF of these cells drift apart following an elongation of the attentional field, the same object stimulates only nine columns that signal a width of nine units (see left part of the figure). Note that an elongation of the attentional field and corresponding RFs shifts along the direction of the lines are sufficient to explain the illusion. That is, the assumption of the field compression in the opposite direction is not necessary but seems biologically plausible

Here, we suggest that such high-level or “global” mechanisms need not be assumed. Rather, the illusion can be explained by changes in low-level processes of spatial coding. The basic idea is illustrated in Fig. 1. We suggest that the illusion arises because the shape of attentional distribution varies somewhat depending on line orientation. In particular, the attentional focus is slightly elongated along the orientation of the lines, which comes with a decrease in density of receptive fields (RFs) along the direction of the lines. Projecting an object of the same size on this distorted mosaic of receptive fields naturally produces the illusion because the line objects activate the receptive fields of fewer neurons along the orientation of the lines.

This idea emerged based on the results of numerous studies demonstrating systematic effects of spatial attention on object appearance (e.g., Fortenbaugh et al., 2011; Gobell & Carrasco, 2005; Liu et al., 2009; Suzuki & Cavanagh, 1997; for a review, see Carrasco & Barbot, 2019). For example, a circular stimulus is perceived as larger when attention is focused on its center than in a neutral attention condition (Anton-Erxleben et al., 2007; Kirsch et al., 2018, 2020). Moreover, the same stimulus is perceived as smaller when the attentional focus is more broadly distributed (Kirsch et al., 2018, 2021; see also Bocianski et al., 2010; Fortenbaugh & Robertson, 2011; Kirsch & Kunde, 2021a, for related findings in location perception). These and similar effects are often explained by assuming that the locations and shapes of RF of cortical neurons are not static and can vary depending on attentional conditions (Anton-Erxleben & Carrasco, 2013; Baruch & Yeshurun, 2014; Carrasco & Barbot, 2019; Kirsch et al., 2018; Kirsch & Kunde, 2021a; Klein et al., 2016; Suzuki & Cavanagh, 1997; for neurophysiological evidence see, e.g., Anton-Erxleben et al., 2009; Klein et al., 2014; Ni et al., 2014; Womelsdorf et al., 2008).

A shift of RFs towards the center of attention, for example, increases the perceived size of the attended object because this object activates neurons with RFs originally located further away from the object’s center or, in other words, because it activates more neurons than when it is unattended (see, e.g., Anton-Erxleben & Carrasco, 2013). A decrease of this RF-shift (or a shift in the opposite direction) can explain the observed decrease in perceived object size with broadly distributed attention (Kirsch et al., 2018; Kirsch & Kunde, 2021a). Previously, we have already suggested that such attentional mechanisms can account for optical illusions, such as the Titchener circles (or Ebbinghaus illusion) and the Roelofs illusion, at least to some extent^1^ (Kirsch, 2022; Kirsch & Kunde, 2021b). Here, we suggest that the same basic mechanisms are responsible for the Helmholtz’s square illusion. In particular, the assumed elongation of the attentional focus along the orientation of the lines is basically an increase of the attentional distribution along this dimension. This should entail a decrease of RF density due to a decrease of RF shifts towards the center of attention (or due to RF shifts in opposite direction) and thus produce an apparent decrease of the respective object dimension in perception.

This attentional explanation allows for testable predictions. The general testbed is to show that manipulation of the shape of the attentional distribution around Helmholtz’s squares affects the magnitude of the illusion. We induced attentional foci which were either similar (or congruent) or dissimilar (incongruent) to (with) the attentional distribution presumably elicited by the line stimuli (see Fig. 1). The main prediction was straightforward. The magnitude of the illusion should decrease in the incongruent as compared with the congruent condition.

## Experiment 1

**Fig. 2:**
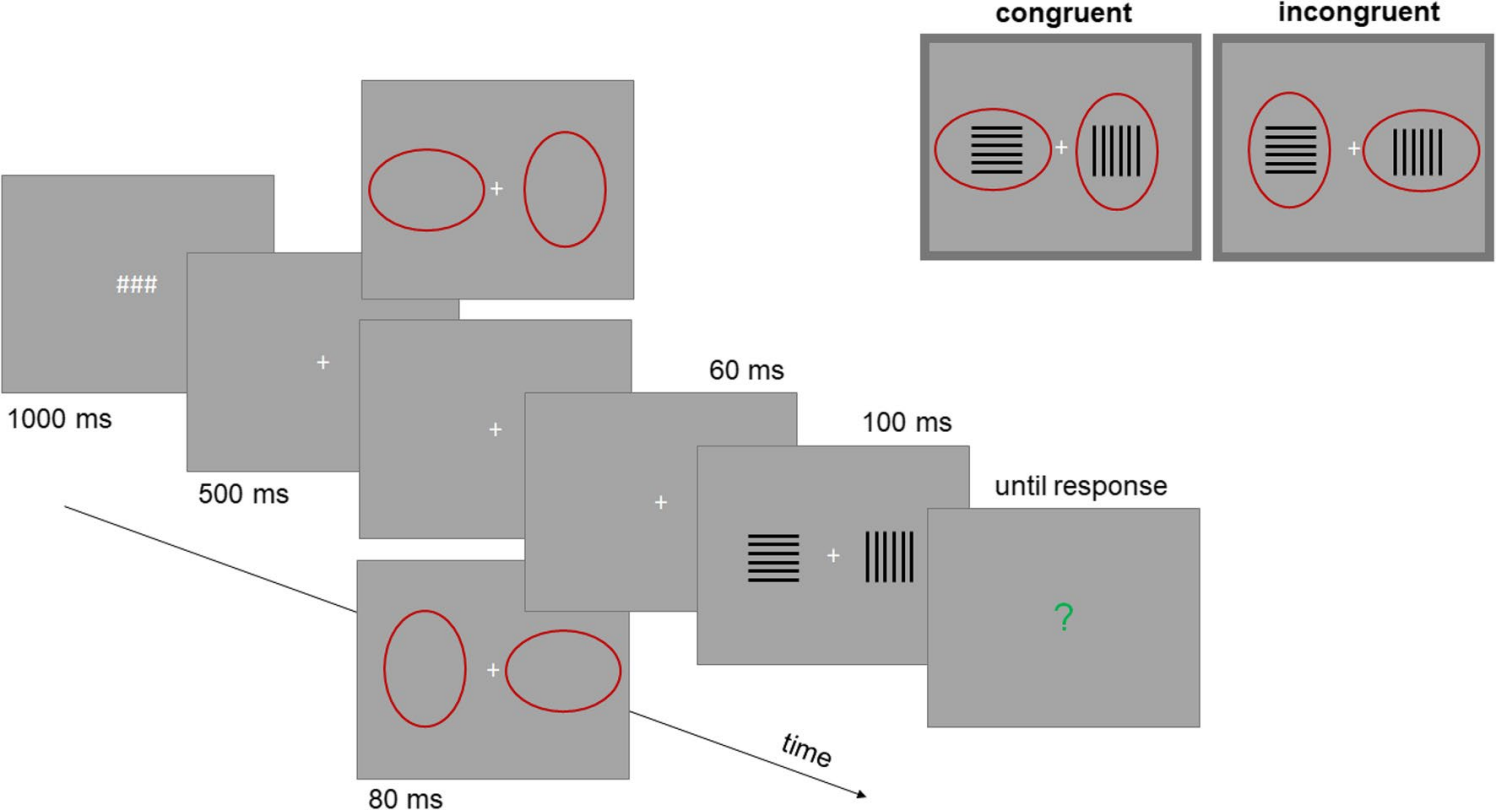
Main trial events in Experiment 1. The crucial spatial relation between attentional cues and targets is outlined in the right upper part. Stimuli shown here are not drawn to scale. (Color figure online)

Figure 2 illustrates a single trial of Experiment 1. To measure the illusion we presented two rectangular target objects including horizontal and vertical lines simultaneously to the left and right of a fixation cross for a short time period and used a method of constant stimuli. One object of a constant size served as a standard stimulus, the other object including varying line lengths served as a test stimulus. Participants were asked to judge either which object is taller (when the test stimulus included vertical lines) or wider (when the test stimulus included horizontal lines; see also Thompson & Mikellidou, 2011). The subjective equality between standard and test stimuli informed about the magnitude of the illusion.

To systematically manipulate the attentional distribution we presented unfilled ovals shortly before the target objects (see also e.g., Kirsch & Kunde, 2021a; Kosovicheva et al., 2010; Yeshurun & Carrasco, 2008). The orientation of these figures was either congruent or incongruent with the orientation of the lines (see upper right part of Fig. 2). The illusion was assumed to decrease for the incongruent condition as compared with the congruent condition.

### Methods

#### Participants

Nineteen psychology students of the University of Würzburg were recruited through the participant-acquisition system (SONA systems). They provided informed consent before participation and received course credit for their participation. The sample included 17 females and two males (age: *M* = 21 years, *SD* = 4). This sample size ensured a power of .80 (*α* = .05) for effect sizes of about *d* = 0.6. The Helmholtz illusion as well as the effects of attentional cues on size perception are robust phenomena that can be demonstrated using few observers (e.g., six in Thompson & Mikellidou, 2011, Experiment 1, or 12 in Kirsch et al., 2018). For example, the mean effect size (*d*_z_) for the effects of cues amounted 2.4 in Experiment 1 of Kirsch et al. (2018), which would require three participants to demonstrate this effect (given a power of .80 and *α* of .05). However, the attentional manipulation studied here has a yet unknown effect size. So assuming a slightly larger than medium effect size appeared to be a reasonable approach.

The study has been approved by the local ethics committee (Ethikkommission des Institutes für Psychologie der Humanwissenschaftlichen Fakultät der Julius-Maximilians-Universität Würzburg, GZEK 2020-88).

#### Apparatus

E-Prime software (Version 3.0; Psychology Software Tools, Pittsburgh, PA) was used to program the experiment and the “E-Prime go” application to run it online. Participants had to download the program files and to perform the experiment on their own computers (running Windows). The spatial resolution of the most screens was 1,920 × 1,080 pixels (15 participants). The remaining four screens had the following resolution: 3,240 × 2,160; 1,366 × 768; 3,840 × 2,160; and 2,560 × 1,440. The refresh rate of all screens was about 60 Hz. We did not have access to the actual sizes of the screens and could thus not calibrate on-screen sizes and distances of stimuli across the participants. We also did not control the participants’ distance from the screen. As a result, the size of the retinal projection of the stimuli could vary across the participants. A potential drawback that comes along with a lack of control of online studies was expected to not substantially limit the results because the crucial experimental manipulations were applied within rather than between the participants.

#### Stimuli

All stimuli were presented on a gray background (with RGB coordinates of 128, 128, 128). Number-sign symbols (###, ~ 18 × 40 pixels; this size would correspond to 0.5 × 1° of visual angle assuming that participants were sitting 57 cm away from the monitor and one pixel measured 0.25 mm) and fixation crosses (7 × 7 pixels/0.2 x 0.2°) were light gray (RGB value: 175, 175, 175). Question marks were presented in green. These stimuli appeared in the center of the screen.

The critical target stimuli were two rectangular objects composed of 10 black lines (thickness = 2 pixels/0.05°) each and presented to the left and to the right of the fixation cross at a distance of 250 pixels (from the center of each object to the center of the screen/6.3°). The lines were oriented either vertically or horizontally with a spacing of 18 pixels (0.5°). One of two objects always contained vertical lines, while the other object always contained horizontal lines.

Two dark red ovals served as attentional cues (250 × 400 pixels/6.3 × 10°). These stimuli were presented at the same locations as the target stimuli (i.e., their centers were 250 pixels/6.3° to the left and to the right of the center of the screen). One of the ovals was always oriented horizontally, while the other oval was always oriented vertically. As a result, the ovals’ contours next to the center of the screen were closer to the center of the screen for the horizontal than for the vertical ovals (see Fig. 2).

#### Trial procedure

Each trial started with three number signs displayed for 1,000 ms followed by a fixation cross that appeared for 500 ms. Then, in some trials, the attentional cues (i.e., ovals) were presented for 80 ms. In some other trials, these cues were omitted and the screen remained blank (except for the fixation cross) for 80 ms (see Design section). Following 60 ms, during which the screen was blank, the target stimuli appeared for 100 ms. The next display contained a question mark and prompted participants to judge either which one of two target objects is “wider” or which one is “taller” (see Fig. 2). Participants responded by pressing the left (for the left target) or the right (for the right target) mouse key. During initial practice trials, feedback was given about whether the response was correct (German words for “correct” [in green] and “a close miss” [in orange] were displayed for 250 ms). During the main experiment, no feedback was given.

#### Design

A method of constant stimuli was applied so that one of the target objects served as a standard stimulus, the other target object served as a test stimulus. Accordingly, if the standard stimulus was composed of horizontal lines, the test stimulus contained vertical lines. The line length of the standard stimulus was always constant and amounted to 162 pixels (4.1°). The overall physical shape of this object was a square (provided the same size of each pixel in the horizontal and vertical dimensions). The length of lines that constituted the test stimulus varied between 77.5 and 122.5% in equidistant steps of 5% in respect to the length of lines that constituted the standard stimulus. Thus, the test stimulus was either wider or narrower when the standard stimulus contained vertical lines, and either higher or shorter when the standard stimulus contained horizontal lines.

The orientations of the ovals varied so that they were either *congruent* with the orientations of the lines or *incongruent* (see Fig. 2, upper right part). We also included a baseline condition in which the ovals were omitted (*no cue*). In one half of trials participants were asked to judge the perceived *width* of the objects, and in the other half of trials they judged the perceived *height* of the objects. In case of width judgments, the standard stimulus always contained vertical lines and the test stimulus always contained horizontal lines. For the height judgments, the opposite was true.

Overall, there were 60 experimental conditions as the factorial combination of “test size” (10) × “cue–target congruency” (3: congruent, incongruent, no cue) × “judgment type” (2: width, height). Each condition was repeated 12 times. The main experiment was divided into four blocks of trials including 180 trials each. In two blocks, width judgments were required whereas in the other two blocks the relative height of the target objects was judged. The order of blocks was randomized. The order of conditions within each block was also randomized.

At the beginning of the experiment, participants performed 24 practice trials (12 × width judgments + 12 × height judgments) in which visual feedback was provided about whether the perceptual judgment was correct or not (see also Trial Procedure section). These trials were not included in the analyses.

#### Data analysis and predictions

For each test stimulus, we computed the proportion of trials in which the test stimulus was judged as wider (width judgments) or taller (height judgments). This was done for each congruency condition. Based on the observed proportion data we estimated psychometric functions using a local model-free fitting procedure (Zychaluk & Foster, 2009) and then determined the so-called points of subjective equality (PSE). The PSE represents a test size (i.e., width or height) at which the proportion of “test stimulus is wider/taller” decisions amounted to 50% and indicates how wide/tall the standard stimulus is perceived as compared with the test stimulus.

**Fig. 3:**
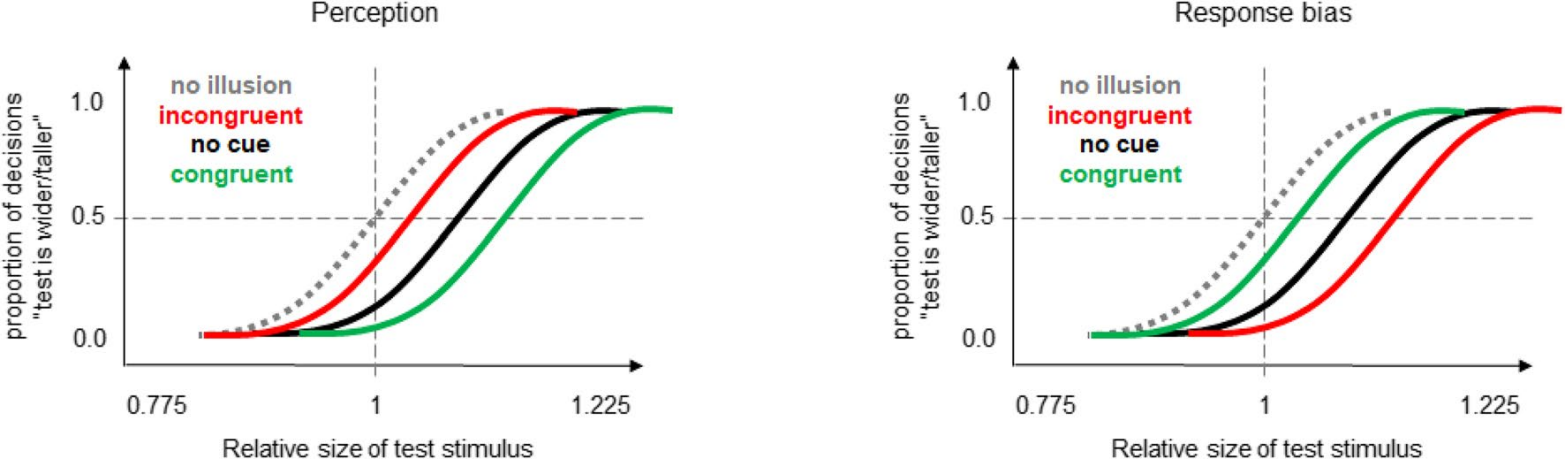
Hypothetical patterns of results predicted based on assumed changes in perception (left part) and on a possible response bias (right part). (Color figure online)

The left part of Fig. 3 shows how the Helmholtz illusion and its possible changes can be measured using this method. If there is no Helmholtz illusion and its changes (i.e., if the length of vertical lines is perceived as equal to the length of horizontal lines and the cues have no impact) then the PSEs should approximate the width and the height of the standard stimulus (i.e., the value of “1” of the test stimulus, see “no illusion” in Fig. 3). If the object with vertical lines is perceived as wider and shorter as the Helmholtz illusion predicts, then the PSEs should shift to larger test stimulus values (i.e., to the right). Importantly, the congruent attentional cues should increase, whereas the incongruent cues should decrease the magnitude of the illusion (i.e., the PSE should increase/decrease for the congruent/incongruent conditions) according to our rationale (see the left part of Fig. 3; see also the Introduction). The main prediction was thus a significant main effect of the factor congruency and a significant difference between the congruent and incongruent conditions.

In theory, the method we used could be susceptible to a response bias in that possible changes in PSE depending on cue–target congruency could occur if the orientation of the cues is taken into account during the judgment instead of or additionally to the perceived width or height of the target objects. In particular, participants could report the location of the wider or taller cue (instead of target) at least in some trials. Although we made an effort to prevent such a behavior by thorough instructions (that stressed that the ovals were irrelevant for the task and should be ignored) and by providing feedback during initial practice trials it could still be present in the collected data. A crucial point here is that this possible response bias should affect the PSE in an opposite way as our attentional hypothesis suggests. That is the PSE should be smaller for the congruent than for the incongruent condition (see Fig. 3, right part). If participants would always report the orientation of the wider/taller cue (i.e., irrespective of the size of the test stimulus), then the psychometric functions of the congruent and incongruent conditions would be flat and would approach the values of “1” and “0” on the *y*-axis, respectively.

### Results and discussion

One participant was excluded from analyses because her/his judgment behavior did not substantially change across the test stimulus in trials including attentional cues so that PSE estimations were not meaningful. We suppose that this participant reported the orientation of the cues in these trials (see Fig. S1 in the supplementary materials and the Data Analysis and Predictions section).

**Fig. 4:**
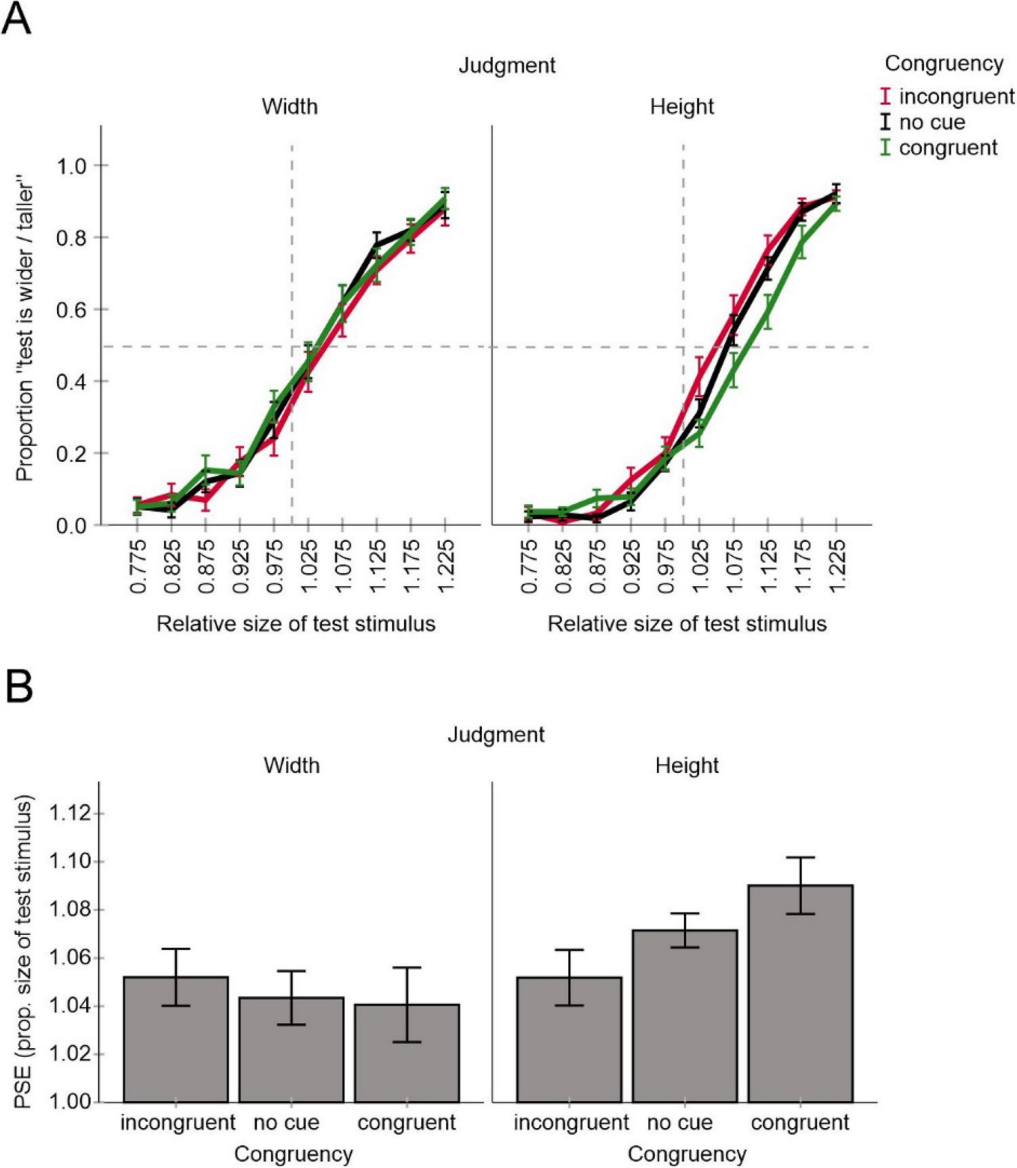
Results of Experiment 1. Shown are mean judgment data for all conditions (**A**) and mean PSE values (**B**). Error bars are standard errors. (Color figure online)

The mean judgment data and the corresponding PSE values of the remaining participants are shown in Fig. 4. All PSE values were significantly larger than one, all *p* < .001 (two-tailed *t* tests against one), indicating that target objects with vertical lines were perceived as wider and shorter than target objects with horizontal lines consistent with the Helmholtz illusion. The magnitude of the illusion varied between 4 and 9% consistent with a previous report (Thompson & Mikellidou, 2011).

An analysis of variance (a two-way ANOVA) including cue–target congruency and judgments type as within subject factors revealed a significant main effect of judgment type, *F*(1, 17) = 7.22, *p* = .016, η_p_^2^ = .298, and a significant interaction, *F*(2, 34) = 6.52, *p* = .004, η_p_^2^ = .277 (other *p* = .4). PSEs were on average larger for height judgments than for width judgments. More importantly, mean PSE increased from incongruent to congruent condition for height judgments as predicted but tended to decrease for width judgments consistent with a response bias (see also Fig. 3). An ANOVA of height judgments including cue–target congruency as a within subject factor revealed a significant main effect of congruency, *F*(2, 34) = 3.81, *p* = .032, η_p_^2^ = .183, while Bonferroni-corrected pairwise comparisons (adjusted α = .0167) revealed no significant differences between congruent, no cue and incongruent conditions respectively (*p*s < .145). An analysis of width judgments did not reveal significant effects, *F*(2, 34) = .70, *p* = .505, η_p_^2^ = .039.

These results suggest that the attentional manipulation had a systematic impact in the predicted direction. However, this effect was relatively weak and evident only when the participants judged the height of the target stimuli, not their width. Moreover, the magnitude of the illusion was on average larger for height judgments than for width judgments. Such an effect has also been reported by Thompson and Mikellidou (2011). The authors reasoned that the vertical–horizontal illusion (i.e., the tendency to perceive vertical lines as longer than horizontal lines of the same length) might be a factor that can account for this outcome on top of the actual Helmholtz illusion (see also Mikellidou, 2012).^2^ This additional distortion can arise because the visual field is somewhat compressed along the horizontal meridian relative to the vertical meridian (e.g., Künnapas, 1957).

## Experiment 2

We wondered about why the results of Experiment1 were only partially consistent with the basic idea shown in Fig. 1 and reasoned that this could be related to the overall distribution of spatial attention in the task we used. If to be attended stimuli are presented in the horizontal dimension as in Experiment1, the spatial attention should generally be distributed along this dimension irrespective of the local attentional cues. Asking to report objects’ width likely strengthened such a horizontal attentional mode. It is thus conceivable that vertically oriented cues could not substantially change this strong sustained horizontal attentional distribution and/or that horizontal cues had less or no impact because attentional focus was already strongly elongated along the horizontal dimension. As a result, no cue effects emerged under these conditions.

In Experiment 2, we tested this assumption presenting target stimuli along the vertical dimension. This should promote a vertically oriented distribution of the attentional focus. As a result, a possible impact of the cues should be diminished or even eliminated when the task requires a focus on objects’ height because horizontally oriented cues would not substantially change this very strong vertical attentional distribution and/or because vertical cues would have less or no impact on an attentional field that is already strongly elongated along the vertical. In other words, the effect of the attentional cues on the illusion should now be observed primarily for width judgments and to a lesser extent, if at all, for height judgments.

### Methods

#### Participants

We aimed to have the same sample size as in Experiment1 (i.e., *n* = 18). After collecting the data from eighteen participants we observed that several of them had to be excluded due to a very low discrimination performance (see Fig. S2 in the supplementary materials and the Results and Discussion section). We thus continued data collection until we had 18 analyzable data sets. Overall, 24 psychology students were recruited through the participant pool (SONA systems) of the University of Würzburg. They provided informed consent before participation and received course credit for their participation. The sample included 16 females and 8 males (age: *M* = 22 years, *SD* = 4).

#### Apparatus

The used software was the same as in Experiment 1. The spatial resolution of many screens was again 1,920 × 1,080 pixels (17 participants). The remaining resolutions were 1,366 × 768 (three participants), 1,600 × 900 (one participant), 2,736 × 1,824 (one participant), 3,000 × 2,000 (one participant), and 1,280 × 800 (one participant). The refresh rate of all screens was about 60 Hz.

#### Stimuli

Stimuli were the same as in Experiment1 except for the locations of attentional cues and target objects. In Experiment 2, these stimuli were presented above and below the fixation cross (at a distance of 250 pixels; cf. Experiment 1).

#### Trial procedure and design

**Fig. 5:**
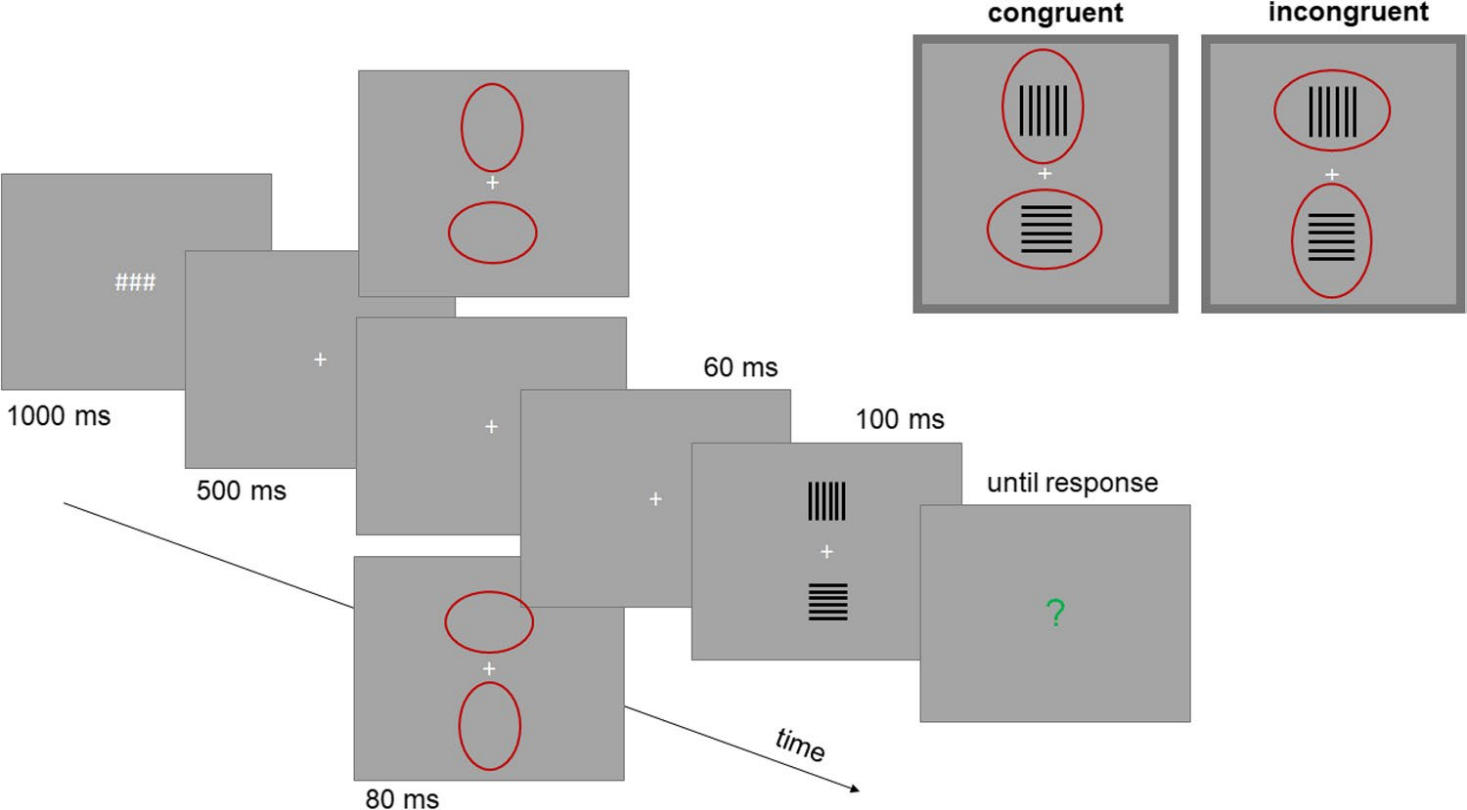
Main trial events in Experiment 2. The crucial spatial relation between attentional cues and targets is outlined in the right upper part. Stimuli shown here are not drawn to scale. (Color figure online)

Trial procedure and design were the same as in Experiment 1, except that participants responded by pressing the upper (for the upper target) or the lower (for the lower target) arrow keys (see Fig. 5).

#### Data analysis and predictions

The data were analyzed in the same way as in Experiment 1. We now predicted a systematic modulation of the illusion (i.e., an impact of attentional cues on PSEs) for the judgments of the width but not for the judgments of the height.

### Results and discussion

Six participants were excluded from analyses because their judgment behavior did not substantially differ across the test stimulus in some or all congruency and judgment type conditions so that PSE estimations were not meaningful. Three of them seemed to report the orientation of the cues, the remaining three seemed to guess under some conditions (see also Fig. S2 in the supplementary materials and the Data Analysis and Predictions section of Experiment 1).

**Fig. 6:**
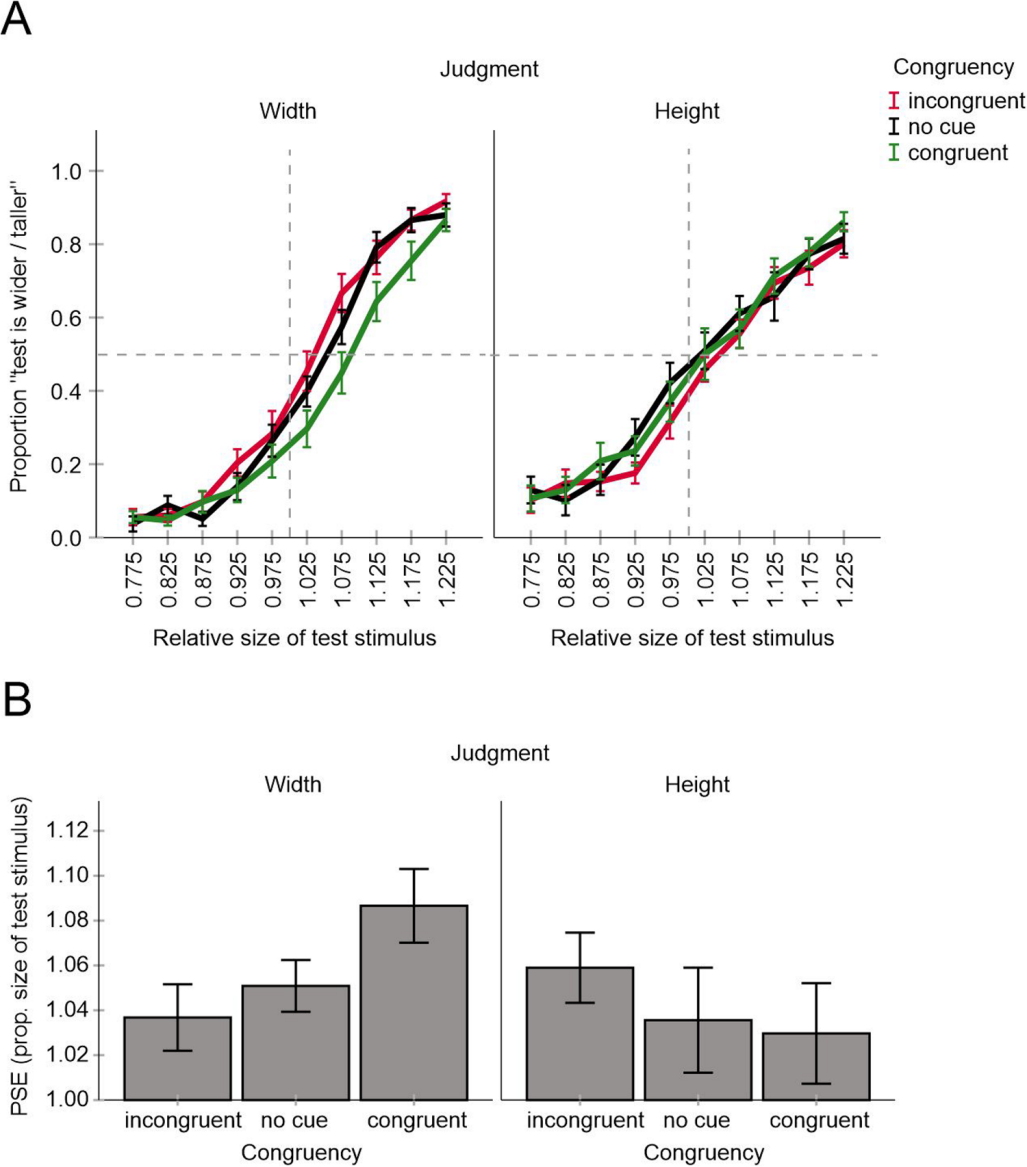
Results of Experiment 2. Shown are mean judgment data for all conditions (**A**) and mean PSE values (**B**). Error bars are standard errors. (Color figure online)

The mean judgment data and the corresponding PSE values of the remaining participants are shown in Fig. 6. All PSE values were significantly larger than one as in Experiment1, all *p*s < .001 (two-tailed *t* test against one), indicating the Helmholtz illusion. The magnitude of the illusion varied between 3% and 9% consistent with the results of Experiment 1.

An analysis of variance (a two-way ANOVA) including cue–target congruency and judgments type as within subject factors revealed a significant interaction, *F*(2, 34) = 7.26, *p* = .002, η_p_^2^ = .299. Neither a main effect of judgment type nor a main effect of cue–target congruency were significant, *F*(1, 17) = .57, *p* = .462, η_p_^2^ = .032, and *F*(2, 34) = 1.06, *p* = .356, η_p_^2^ = .059. Mean PSE increased from incongruent to congruent condition for width judgments as predicted but tended to decrease for height judgments consistent with a response bias (see also Fig. 3). An ANOVA of width judgments including cue–target congruency as a within subject factor revealed a significant main effect for congruency, *F*(2, 34) = 6.98, *p* = .003, η_p_^2^ = .291. Bonferroni-corrected pairwise comparisons (adjusted α = .0167) indicated significant differences of the congruent condition to the incongruent condition, *p* = .024, and to the no cue condition, *p* = .015, while the difference between congruent and no cue condition was not significant, *p* = .883. An analysis of height judgments did not reveal significant effects, *F*(2, 34) = 1.96, *p* = .156, η_p_^2^ = .103.

These results suggest that the attentional manipulation had a systematic impact only when the participants judged the width of the target stimuli, not their height. This pattern is consistent with what we predicted based on the results of Experiment 1. In contrast to the results Experiment 1, the illusion was not more strongly pronounced for height judgments than for width judgments. Descriptively, the trend was even in the opposite direction. This could be a clue that the impact of the vertical–horizontal illusion decreased when attention was distributed along the vertical dimension (see also Results and Discussion section of Experiment 1). This would be in line with the claim that the vertical–horizontal illusion arises because the visual field is usually extended along the horizontal dimension (e.g., Künnapas, 1957). The author demonstrated that this illusion decreases (increases) when the lines are surrounded by a vertical (horizontal) ellipse, or in our terms, following a variation of attentional distribution.

## Experiment 3

The experimental logic of Experiment1 and Experiment 2 rests on the assumption that the shape of the attentional focus adapts to the shape of the attentional cue (see Experiment1). Although plausible (see also e.g., Kirsch & Kunde, 2021a; Kosovicheva et al., 2010; Yeshurun & Carrasco, 2008) this assumption is not necessarily valid. In other words, the effects observed in Experiment 1 and Experiment 2 could reflect a certain impact of cues on judgment behavior, but not necessarily of spatial attention. Another possible limitation is that the experiments were performed in an online format that goes along with a lack of experimental control (esp. over the size of the retinal projection of stimuli; see also Experiment 1). Experiment 3 was done to evaluate these potential concerns.

**Fig. 7:**
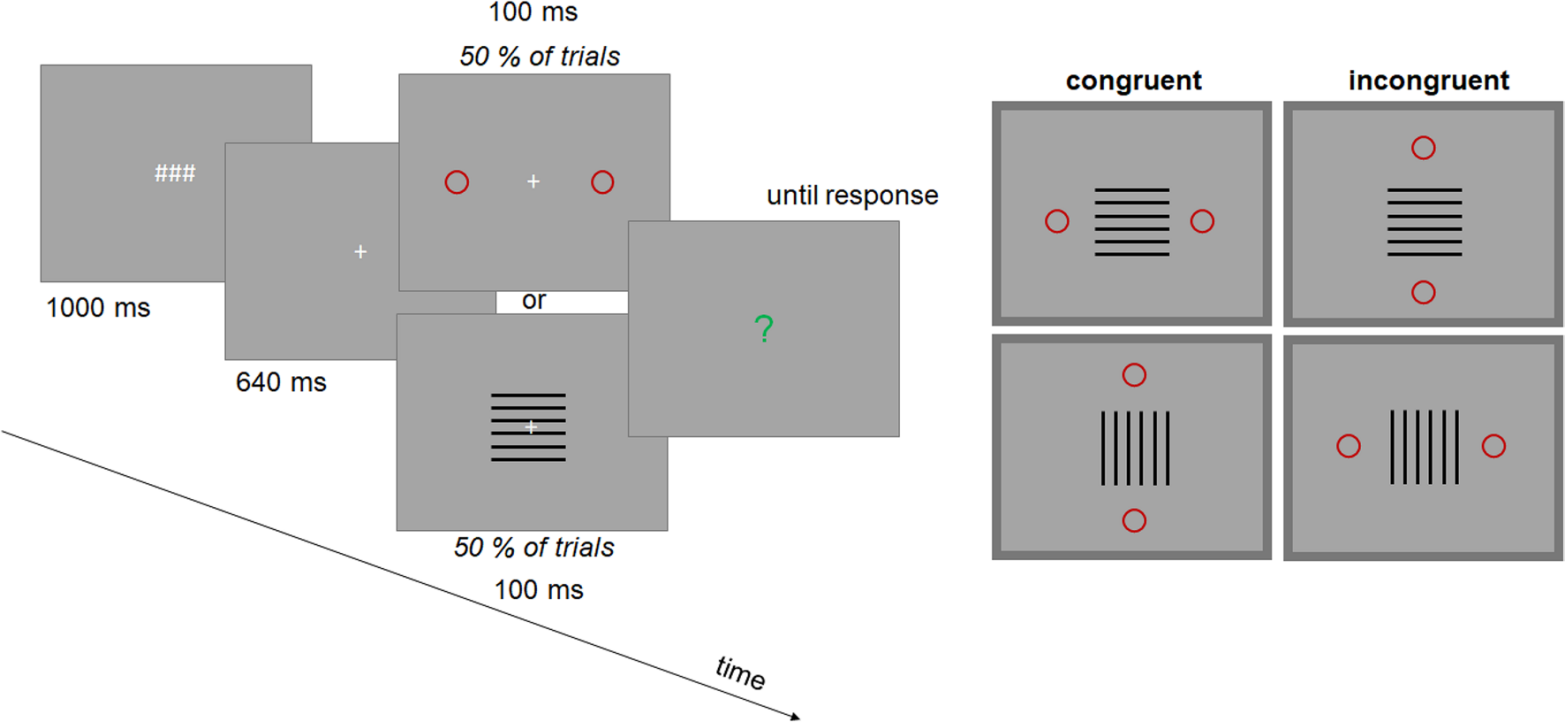
Main trial events in Experiment 3. The crucial spatial relation between attentional cues (i.e., circles) and targets (rectangular line objects) is outlined in the right part. Stimuli shown here are not drawn to scale. Note, circles and line objects were presented in different trials of a block, but not simultaneously. The right panel illustrates the four possible combinations of circle placement and line orientation in each of the four types of blocks, in which the assumed spread of visual attention induced by circles and line orientation of the rectangular objects were either congruent or incongruent. (Color figure online)

We now omitted attentional cues and induced sustained rather than transient changes in the distribution of spatial attention by using a secondary task in a laboratory setting. Participants either judged whether a rectangular target object is taller or wider than a square (in one half of trials) or they compared the sizes of two smaller circles (in another half of trials) within a single block of trials. Both tasks were thus performed in close temporal proximity but in separate trials. The target object was presented in the center of the screen and the spatial positions of the circles were to the left and right of or above and below the center of the screen. In each block, the orientation of the target stimulus (horizontal or vertical) and the locations of the circles in the secondary task (i.e., left and right or above and below the center) remained the same. The rationale was as follows. When the circles appeared left and right of (above and below) the center of the screen the attentional focus was assumed to be elongated along the horizontal (vertical) dimension to comply with the requirements of the secondary task. By analogy to the previous experiments, we speak of a “congruent” condition when this attentional distribution matched the attentional distribution presumably elicited by the line stimuli and of “incongruent” condition when this was not the case (see Fig. 7; cf. also Fig. 1).^3^ If distribution of spatial attention is in fact a source of the Helmholtz illusion as we suggest (see Introduction) then the illusion should decrease in the incongruent as compared with the congruent condition. Such an effect should not arise if the main results of Experiment 1 and Experiment 2 have nothing to do with attention and/or are due to a lack of experimental control.

### Methods

#### Participants

As effects in endogenous attentional tasks are usually smaller than in exogenous tasks, we decided to collect more data than in Experiment 1 and Experiment 2. The sample size was determined a priori to be 24 and ensured a power of .80 (*α* = .05) for effect sizes of about *d* = 0.53. Participants were recruited through the participant pool (SONA systems) of the University of Würzburg. They provided informed consent before participation and received monetary compensation (10 Euro) for their participation. The sample included 18 females and six males (age: *M* = 28 years, *SD* = 6).

#### Apparatus

Experiment 3 was performed in a normally illuminated laboratory. Participants sat in front of a 21.5-in. monitor (Acer G226HQL; 1,280 × 1,024 pixels; 1 pixel = 0.25 mm; 60 Hz) placed at about 53 cm distance. A chin rest was used to support their heads. E-Prime software (Version 3.0; Psychology Software Tools, Pittsburgh, PA) was used to program and to run the experiment.

#### Stimuli

The target stimulus was a rectangular objects composed of 10 either vertically or horizontally arranged black lines as in Experiments 1 and 2 (thickness = 0.05°). It was presented in the center of the screen. The length of the lines varied (see Design). The secondary task contained two dark red circles. These stimuli were presented 5.4° either to the left and to the right of the center of the screen or above and below it. One of the circles was always 0.5°in diameter, while the other circle was 30% smaller in one half of trials and 30% larger in the remaining trials. All other stimuli were the same as in Experiments 1 and 2.

#### Trial procedure

Each trial started with three number signs displayed for 1,000 ms followed by a fixation cross that appeared for 640 ms. Then, in 50% of all trials, the rectangular target object that was composed of vertical or horizontal lines appeared for 100 ms. In the remaining trials, two circles were presented for the same duration (i.e., 100 ms). The next display contained a question mark and prompted participants to judge either whether the target object is taller (left mouse key) or wider (right mouse key) than a square or which one of two circles is larger (one of four arrow keys according to the location of the larger circle) depending on which stimulus was presented before. That is there were no explicit cues indicating the required type of judgment (when the line object appeared, participants had to judge whether it is taller or wider than a square, when the circles appeared, they judged which one is larger). The mouse keys were pressed with the dominant hand, the arrow keys with the non-dominant hand. This was done to enable a better mental separation of both tasks for the participants.

During initial practice trials, feedback was given in both circle and rectangle tasks about whether the response was correct. During the main experiment, no feedback was given in the rectangle task. In the circle task, participants still received feedback if their response was incorrect. Moreover, error feedback was presented and the trial repeated (during practice as well as during the main experiment) when participants intermixed the tasks (i.e., when they pressed a mouse key instead of an arrow key or vice versa).

#### Design

The length of lines that constituted the target stimulus varied between 72.5 and 127.5% in equidistant steps of 5% in respect to the length of lines that constituted a true square (4.4°). This went along with the variation of target shape from 27.5% taller than wider to 27.5% wider than taller. The locations of the circles varied so that attending them was either *congruent* with the orientations of the lines in the target stimulus and thus with the presumed distribution of attention induced by this stimulus or *incongruent* (see Fig. 7, right part).

There were four different types of blocks. Each block type included one of four main experimental conditions resulting from the factorial combination of congruency and orientation of the lines in the target stimulus (congruent & horizontal lines; congruent & vertical lines; incongruent & horizontal lines; incongruent & vertical lines). Each block type was repeated twice in a random order in the course of the experiment (i.e., there were eight blocks of trials). Each block included 60 trials with a line target and 60 target trials with circles. These trials were intermixed and were presented in a random order with the constraint that the type of task could be immediately repeated not more than one time before the next alternation (this was done to constantly maintain attention on both tasks). In each block, each size of the line target was repeated 5 times (random order). Overall, each participant performed 960 trials, 480 line target trials (10 repetitions of each target size in each congruency and orientation condition), and 480 circle trials (120 repetitions in each congruency and orientation condition). At the beginning of the experiment, participants performed 48 practice trials (12 trials for each congruency and orientation condition). These trials were not included in the analyses.

#### Data analysis and predictions

**Fig. 8:**
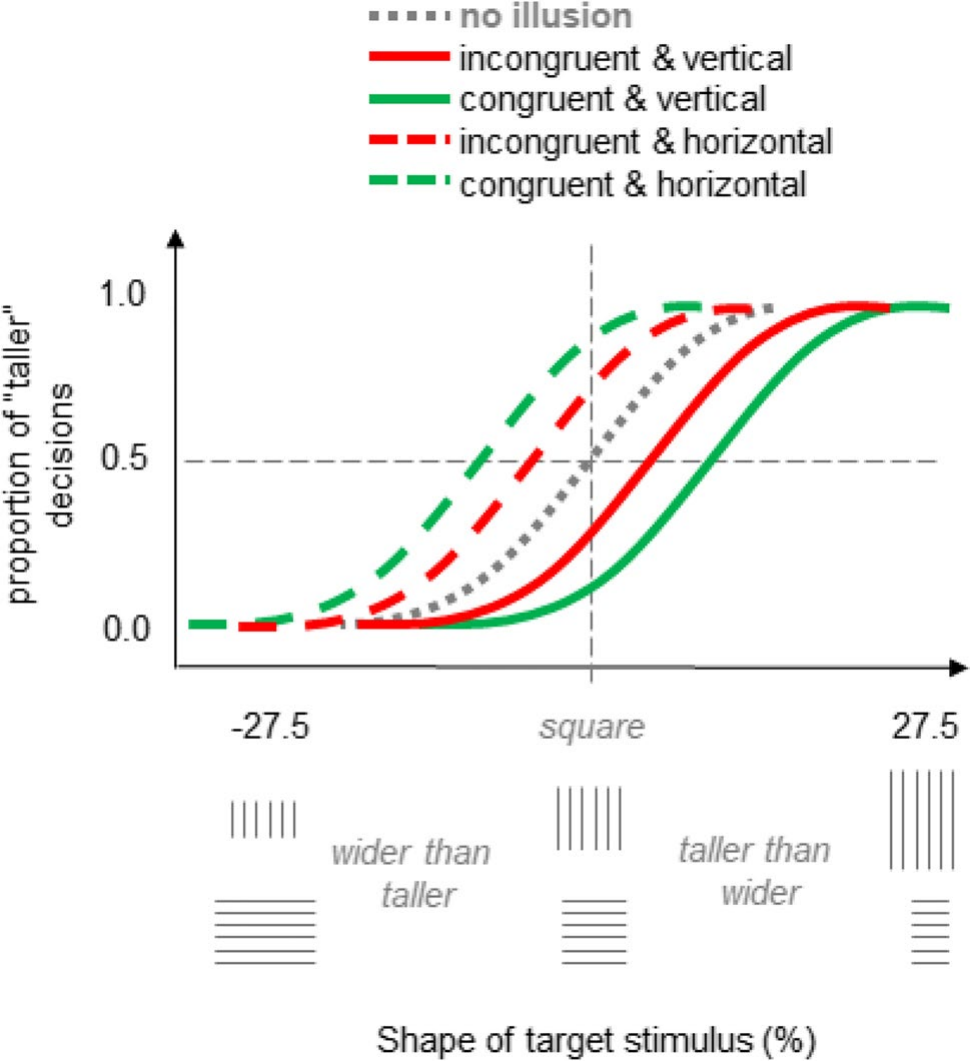
Hypothetical pattern of results for Experiment 3 predicted based on assumed attentional changes in perception. (Color figure online)

For each size of the target stimulus, we computed the proportion of trials in which the target stimulus was judged as taller than a square. This was done for each congruency and each line orientation condition, and each length of the lines (i.e., for each shape of the target). We then determined the PSEs, which indicated the target shape being perceived as a true square. The Helmholtz illusion should be expressed here by a PSE corresponding to a taller than wider shape for vertically oriented lines, and to a wider than taller shape for horizontally oriented lines. Accordingly, for the congruent conditions this shape difference should be larger than for the incongruent conditions (see Fig. 8).

### Results and discussion

Two participants were excluded from analyses because their judgment behavior did not substantially differ across the shapes of the target stimulus in some or all congruency and line orientation conditions in the line target task so that PSE estimations were not meaningful (see also Fig. S3 in the supplementary materials).

#### Circle task

The accuracy in the circle task was on average 98.2% (*SD* = .03) and varied slightly with the location of the circles: It was higher for horizontal (98.6%, *SD* = .02) than for vertical (97.7%, *SD* = .03) circle arrangements, *t*(21) = 2.30, *p* = .032 (*t* test for dependent samples, α = .05). In a two-way ANOVA including congruency and line orientation as factors, this effect was expressed in a significant interaction between congruency and line orientation (see Fig. 7), *F*(1, 21) = 5.27, *p* = .032, η_p_^2^ = .201. The main effects were not significant, *F*(1, 21) = .05, *p* = .827, η_p_^2^ = .002 (congruency), and *F*(1, 21) = .86, *p* = .363, η_p_^2^ = .039 (line orientation).

These results indicate that participants attended to the secondary task and thus adopted their attentional focus accordingly. A better performance for horizontal stimuli is consistent with several studies indicating that for a given eccentricity performance in numerous visual tasks is better along the horizontal than the vertical meridian of the visual field (e.g., Barbot et al., 2021).

#### Line target task

**Fig. 9:**
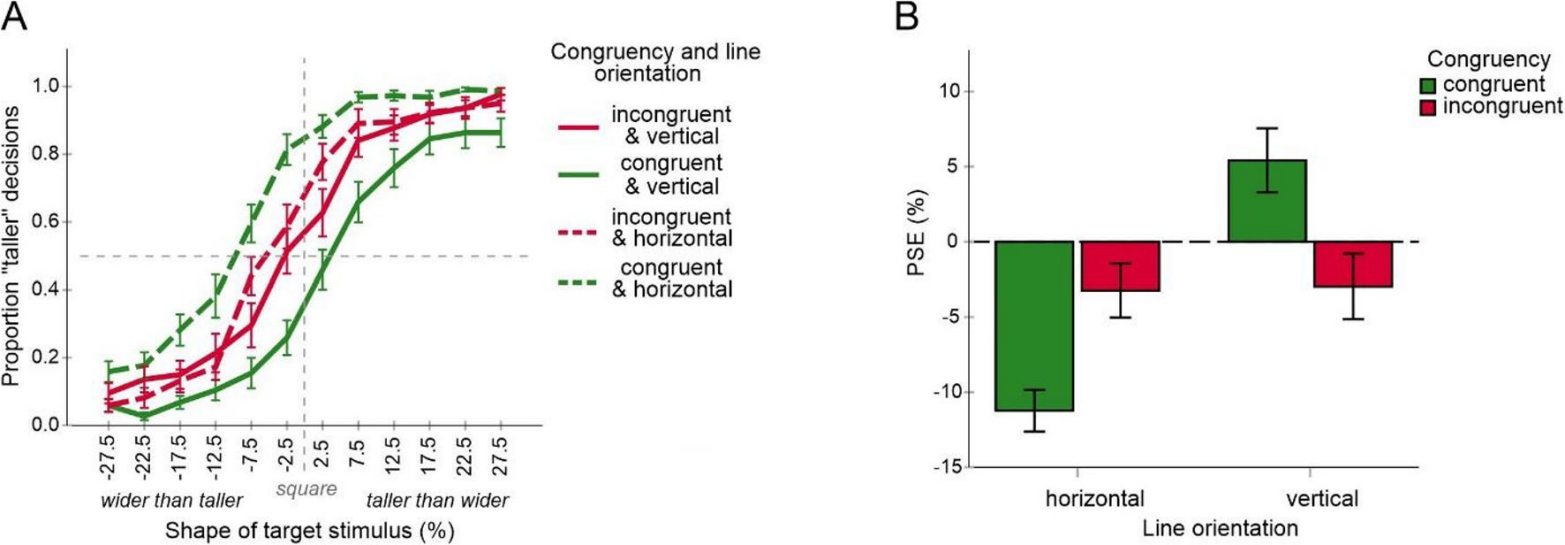
Results of Experiment 3. Shown are mean judgment data for all conditions (**A**) and mean PSE values (**B**). Error bars are standard errors. (Color figure online)

The mean judgment data and the corresponding PSE values are shown in Fig. 9. A two-way ANOVA including congruency and line orientation as factors revealed a significant main effect of line orientation, *F*(1, 21) = 12.79, *p* = .002, η_p_^2^ = .379, and more importantly, a significant interaction, *F*(1, 21) = 41.39, *p* < .001, η_p_^2^ = .663. The PSEs were on average smaller for the horizontal target objects. Crucially, the PSE difference between the congruent conditions was substantially larger, 17.7%, *t*(21) = 7.08, *p* < .001, *t* test for dependent samples, α = .05, than the difference between the incongruent conditions, 0.3%, *t*(21) = .09, *p* = .929, *t* test for dependent samples, α = .05, as predicted. The Helmholtz illusion was observed in the congruent conditions (11.2% and 5.4% in size for the horizontal and vertical lines respectively, both values are significantly different from zero, *p*s < .02, *t* tests against zero, α = .05) and it was basically absent in the incongruent conditions (3.2% and 3.0%, both values are not significantly different from zero, *p*s > .08, *t* tests against zero, α = .05).

This pattern of results suggests that the manipulation of sustained attention had a systematic impact on judgment behavior consistent with our main hypothesis and with the impact of transient attentional cues observed in Experiment 1 and Experiment 2. In particular, the magnitude of the illusion decreased when attention was distributed contrary to the orientation of the lines (i.e., of attentional focus supposedly induced by the lines) as compared with when it was distributed along the lines. Thus, the results speak for that the results of Experiment 1 and Experiment 2 in fact reflect attentional influences as we supposed and are not due to some factors related to a lack of experimental control.

It is worth noting that the illusory effect was substantially larger for horizontal targets than for vertical targets. This result again points to an additional impact of the vertical–horizontal illusion that was obviously present in Experiment 1 but not in Experiment 2. That is, the Helmholtz illusion is weaker for vertical targets because vertical lines are usually perceived as longer than horizontal lines and this counteracts the actual Helmholtz illusion. We reasoned that such an impact could disappear with a strong vertical distribution of attention (see Experiment 2). This could have had an influence on the “asymmetrical” pattern of results of Experiment 3 (cf. Fig. 8 and Fig. 9A) in addition to the orientation of the lines. For example, in the congruent & vertical condition (with vertical distribution of attention) the vertical–horizontal illusion can be assumed to have less impact than in the congruent & horizontal condition (with horizontal distribution of attention). Accordingly, the illusory Helmholtz effect is reinforced in the latter condition, but rather unaffected in the former condition. Also, the trend towards the opposite of the Helmholtz illusion in the incongruent & vertical condition might be basically a trend towards the vertical–horizontal illusion (after reduction of the actual Helmholtz illusion) facilitated by the horizontal attentional distribution.

## General discussion

Numerous stimulus patterns are perceived in a way that differs from what can be expected from the physical reality. Here we focused on one of such patterns known as the Helmholtz square illusion that did not receive much attention so far. A square composed of vertical or horizontal lines appears elongated in perception in the direction opposite to the line orientation. Based on recent research on the interplay between perception and spatial attention we suggest that this perceptual distortion emerges because of changes in attentional distribution, which entail changes in low-level spatial coding (see Fig. 1).

Three experiments were conducted to test this assumption. In Experiment 1 and Experiment 2, transient cues were used to systematically vary attentional distribution. We elicited attentional fields that were congruent or incongruent with the attentional fields supposedly caused by the Helmholtz’s squares. In the congruent conditions, we observed a larger magnitude of the illusion than in the incongruent conditions. This predicted pattern was, however, only evident for judgments of objects’ height when target stimuli were presented horizontally (Experiment 1) and for judgments of objects’ width when target stimuli were presented vertically (Experiment 2). We assume that the lack of the predicted effect in the remaining conditions was due to a strong sustained allocation of attention along the horizontal/vertical dimension when the task was to judge objects’ width/height (Experiment 1/Experiment 2). Consequently, the attentional cues were unable to change this “default” mode of attention substantially.

In other words, the interaction between attention and perception in Experiments 1 and 2 was more complex than we initially assumed. The idea shown in Fig. 1 focusses on “transient” attentional changes caused by the target stimulus and ignores possible impacts of more sustained distributions of attention. In reality, such a sustained attentional mode (and associated changes in RF density) could enhance and reduce the impact of transient attentional processes induced by certain stimuli. Some additional aspects of the results of Experiments 1 and 2 can also be interpreted along this direction. In particular, the magnitude of the illusion was larger for height judgments (than for width judgments) in Experiment 1 but tended to be smaller in Experiment 2 when only no cue conditions are considered. This could indicate that attentional focus was already substantially elongated along the target positions (that were predictable) before the target stimulus was presented so that the objects’ lines had a lesser impact than they could potentially have when attention were more evenly distributed across the visual field.

The results of Experiment 3 strengthen this reasoning. In this experiment, we implemented sustained rather than transient changes of spatial attention. These attentional changes entailed changes in the magnitude of the illusion consistent with the effects observed with transient cues as well as with the suggested explanation for why these effects did not occur in some conditions of Experiment 1 and Experiment 2. As Experiment 3 was performed in a laboratory setting with a strong experimental control, its results also indicate that the main results of Experiments 1 and 2 were not due their online formal that entailed far less experimental control. Overall, the present results suggest that spatial attention substantially contributes to the Helmholtz illusion consistent with the idea outlined in Fig. 1.

Visual illusions are often explained by an inappropriate attempt of the visual system to apply three-dimensional interpretations to two-dimensional images (such as size-constancy scaling; e.g., Gregory, 1963). Another approach emphasized low-level interactions between neural representations of texture’s contours (e.g., Jaeger, 1978) or the spatial frequency of observed objects and its analyses (e.g., Ginsburg, 1984). Also, a kind of fixed distortion in the visual field such as its oval shape was held responsible for perceptual differences between vertical and horizontal objects’ features in some illusions (e.g., Künnapas, 1957). In the context of the Helmholtz’ squares, none of these theories appears plausible. For example, since the square with horizontal lines is a 90° rotated version of the square with vertical lines and the illusory effect changes with rotation (the lines and spacing appear to change in the same direction) a certain vertical–horizontal anisotropy of the visual field cannot account for the Helmholtz’ illusion as a whole (although it can explain why it can be somewhat smaller or larger under certain conditions; see Results and Discussion sections). In a similar vein, theories based on inappropriate three-dimensional interpretations of two-dimensional images would predict an overall increase or decrease of perceived square size rather than a change of perceived width or height. If, for example, the square with vertical lines is perceived as more distant than the square with horizontal lines, then the width as well as height of the square with vertical lines should be perceived as larger. Also, any differences in spatial frequency or in the processing of texture’s contours cannot exclusively explain the illusion because the squares are identical with respect to these types of features.

The idea that spatial attention contributes to visual illusions in not new. Previous research already suggested that certain features of the attentive field constitute several illusions, such as the parallel lines, Ponzo, Mueller-Lyer and Ebbinghaus illusions (e.g., Jordan & English, 1989; Pressey & Murray, 1976; Pressey & Pressey, 1992; Shulman, 1992). Our approach can be considered as a version of this general idea that is also related to the spatial frequency coding assumption (Ginsburg, 1984; see above) and that we applied to the Helmholtz squares. It is rather pragmatic as a logic previously approved in the context of the studies on the interplay between attention and perception was applied to explain the Helmholtz illusion. In particular, why attentional distribution should be elongated along the lines as suggested is not clear at present. One clue for why this could be so can be derived from the Gestalt principle of good continuation indicating a tendency of observers to group stimulus elements in perception so that they form smooth and unbroken contours. Applied to the Helmholtz’s squares, this principle is related to the direction of each line and indicates a perceptual tendency to continue each line along its direction (Pinna, 2015). Such a tendency could be a reason for why attentional focus is elongated along the local spatial orientation of the lines. Importantly, though, our study reveals the distribution of visual attention as the actually causal factor here, rather than gestalt principles per se. This claim as well as the suggested idea in general are of course preliminary, and more research is needed to better evaluate their explanatory and predictive values.

### Supplementary Information

ESM 1 (DOCX 1100 kb)

## Footnotes

1 In the Roelofs illusion, an object is perceived as displaced in the direction opposite to the current locus of attention. This presumably occurs because of the RF’s shifts towards that locus. In the Ebbinghaus illusion, an object surrounded by smaller objects is perceived as larger than when it is surrounded by larger objects. We suggested that this effect is partly due to different sizes of the attentional focus and resulting differences in RF shifts.

2 The basic idea here was that for height judgments the vertical-horizontal illusion acts in the same direction as the Helmholtz illusion. Accordingly, the illusory effect (i.e., a perceptual overestimation of the vertical dimension) is reinforced. For width judgments, in contrast, the vertical–horizontal illusion counteracts and thus reduces the Helmholtz illusion. We agree that such an account can well explain a smaller illusory effect for stimuli with vertical lines (e.g., when the standard stimuli are considered in isolation in Exp. 1). For the present setup, however, it does not appear to be sufficient, at least not without additional assumptions (e.g., if the vertical–horizontal illusion is assumed also to affect the test stimuli, then a decrease rather than an increase of the overall illusory effect is expected for height judgments).

3 We would like to thank Dr. Kyriaki Mikellidou for helpful suggestions regarding the design of this experiment.
