# Supplementary material for: On the origin of the Helmholtz’s square illusion: An attentional account
Source: Atten Percept Psychophys. 2023 May 8;85(6):2018–32. doi: 10.3758/s13414-023-02717-1 (PMC10545586; doi:10.3758/s13414-023-02717-1)
Supplement: Supplementary file 1 — (DOCX 1100 kb) [file 13414_2023_2717_MOESM1_ESM.docx]

**Supplementary materials**

**
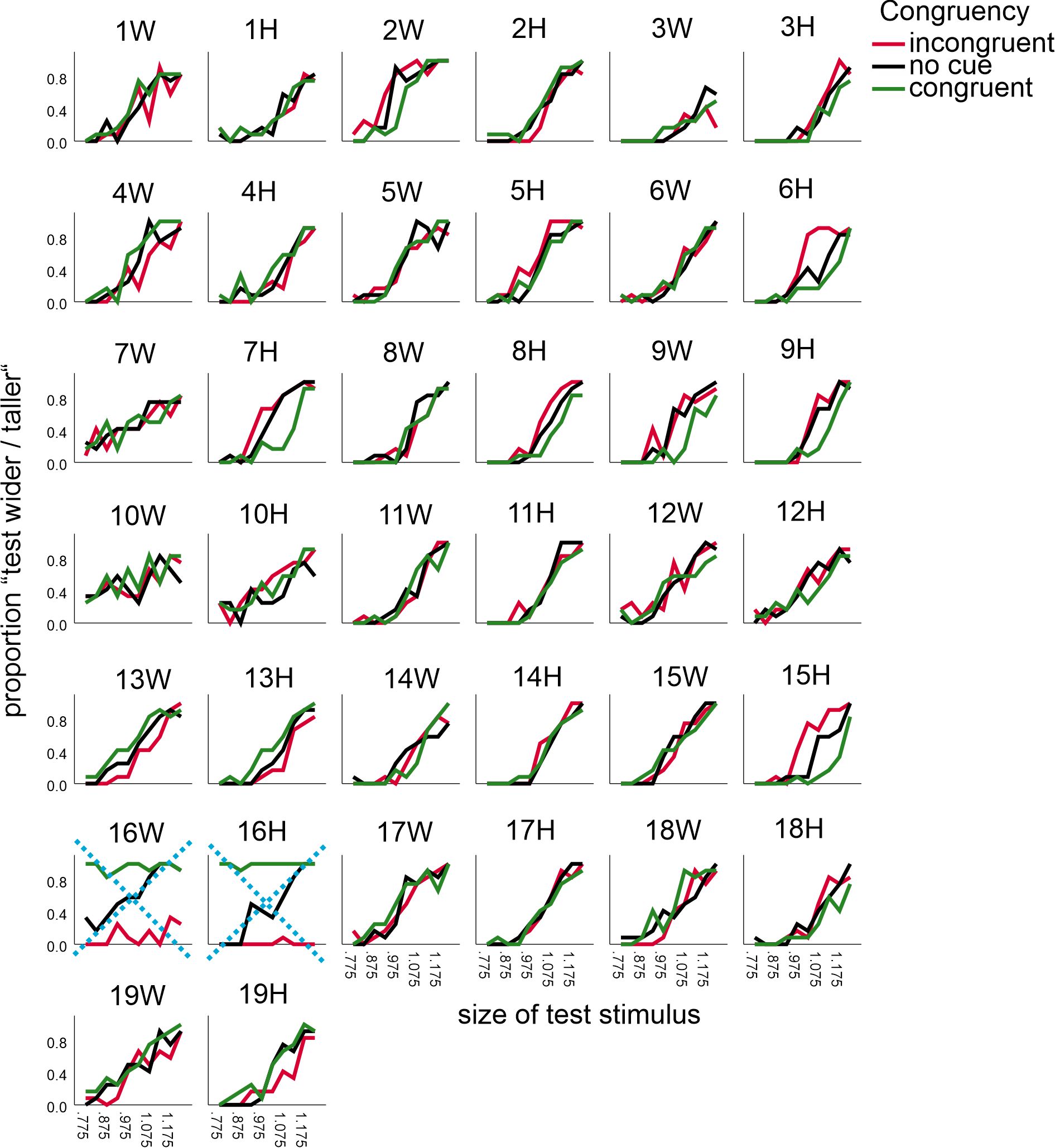
**

***Figure S1****. Individual* *data for Experiment 1. Values indicate the proportion of trials in which the test stimulus was judged as wider or taller than the standard stimulus as a function of cue-target congruency and type of judgment (W = width, H = height). X indicates a participant with low discrimination performance who was not included in the analyses.*

***
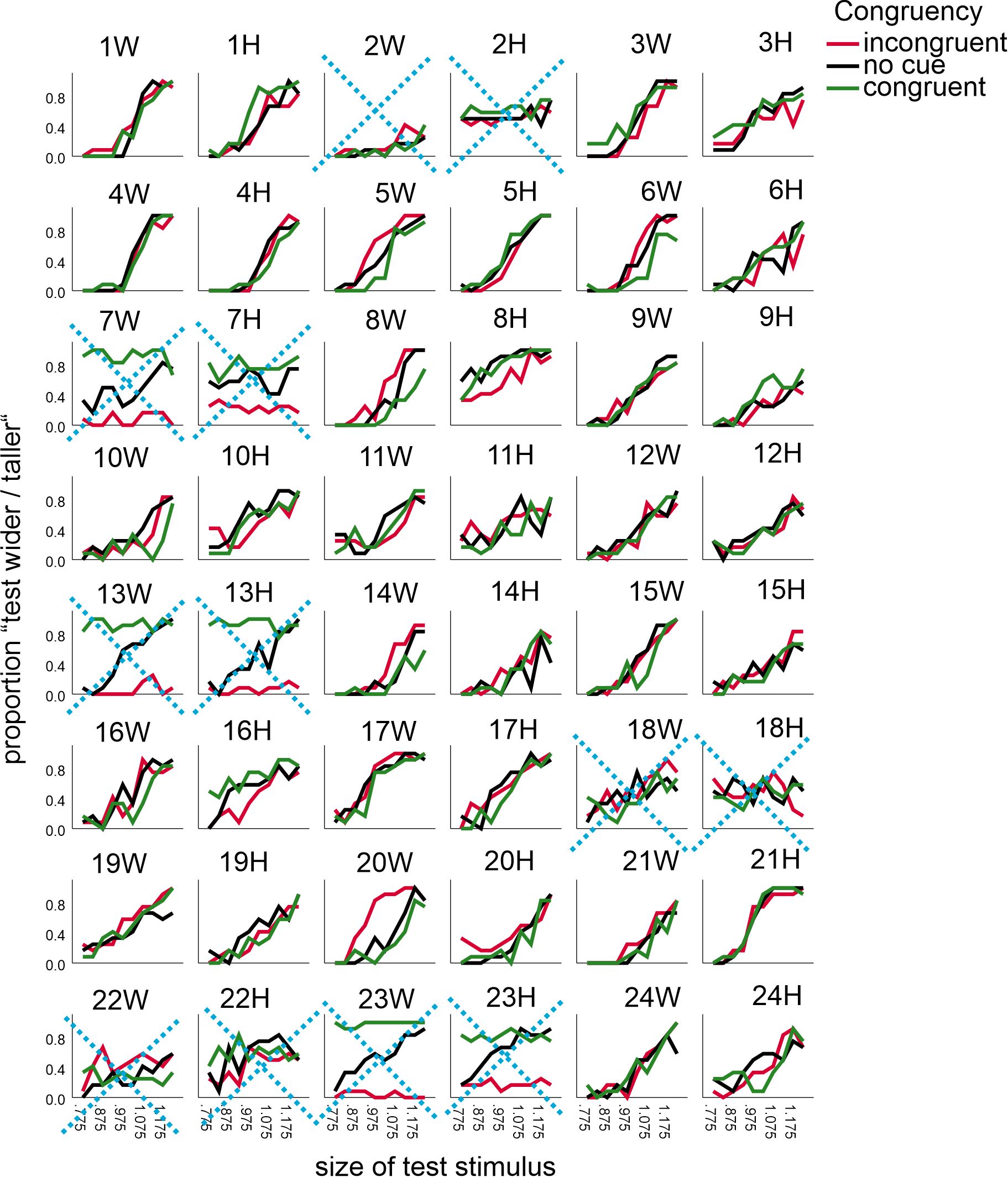
***

***Figure S2****.* *Individual* *data for Experiment 2. Values indicate the proportion of trials in which the test stimulus was judged as wider or taller than the standard stimulus as a function of cue-target congruency and type of judgment (W = width, H = height). Xs indicate participants with low discrimination performance which were not included in the analyses.*

***
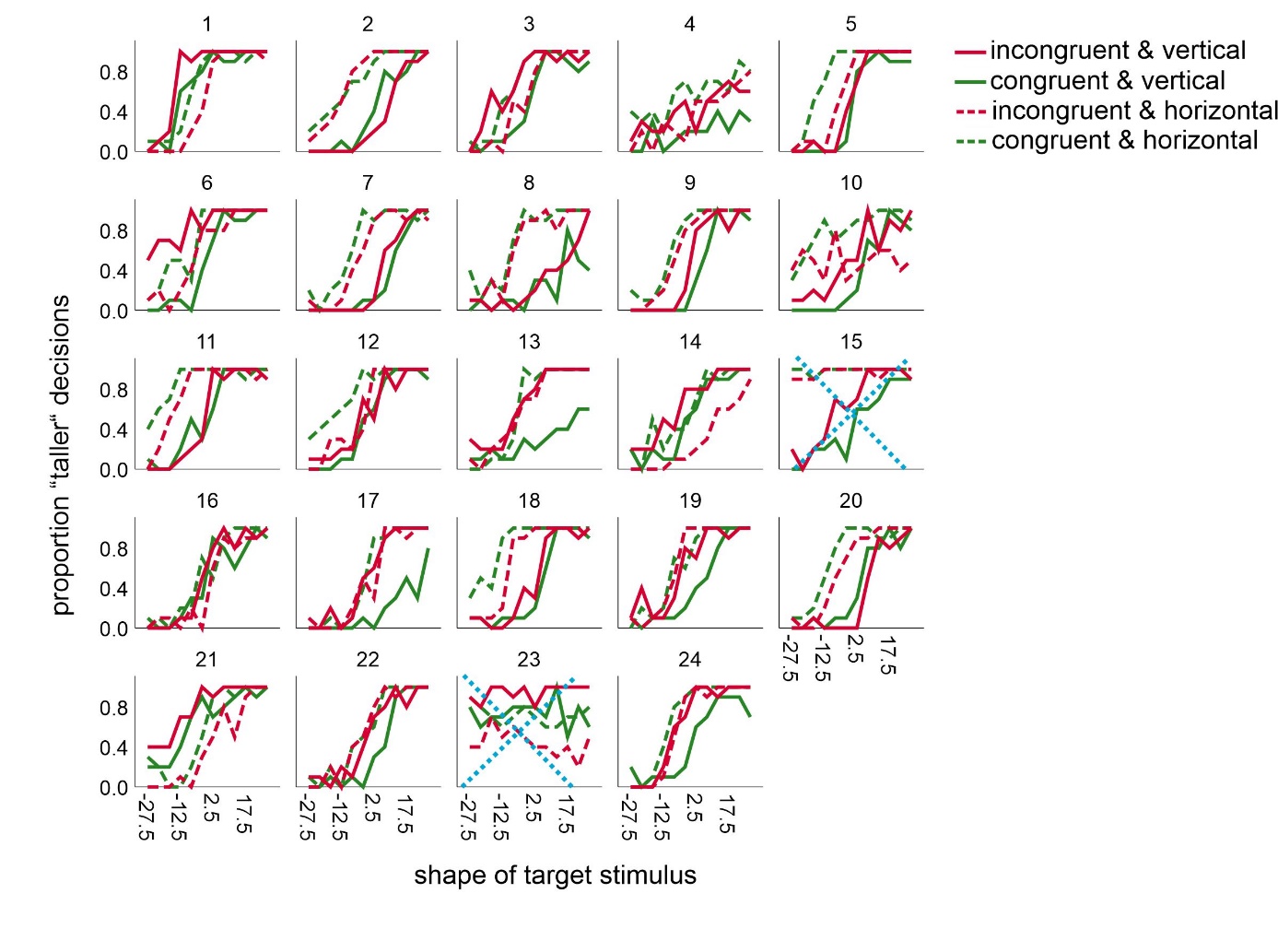
***

***Figure S3****. Individual* *data for Experiment 3. Values indicate the proportion of trials in which the target stimulus was judged as taller than a square as a function of congruency and line orientation. Xs indicate participants with low discrimination performance who were not included in the analyses.*
